# The complete chloroplast genome sequence of *Cynanchum forrestii* Schltr. (Asclepiadaceae) and its phylogenetic analysis

**DOI:** 10.1080/23802359.2019.1678437

**Published:** 2019-10-21

**Authors:** Jie Zhang, Dequan Zhang

**Affiliations:** a College of Pharmacy and Chemistry, Dali University, Dali, PR China;; b Institute of Materia Medica, Dali University, Dali, PR China;; c Key Laboratory of Yunnan Provincial Higher Education Institutions for Development of Yunnan Daodi Medicinal Materials Resources, Yunnan, PR China

**Keywords:** *Cynanchum forrestii*, medicinal plant, complete chloroplast genome, phylogenetic analysis

## Abstract

*Cynanchum forrestii* is a folk medicinal plant in southwest China. In this study, we sequenced complete chloroplast (cp) genome sequence of *C. forrestii* to investigate its phylogenetic relationship. The whole cp genome of *C. forrestii* was 159,917 bp in length with 43.5% overall GC content, including a large single-copy (LSC) region of 91,189 bp and a small single-copy (SSC) region of 19,972 bp, which were separated by a pair of inverted repeats (IRs) of 24,378 bp. The cp genome contained 112 genes, including 78 protein-coding genes, 30 tRNA genes, and 4 rRNA genes. The phylogenetic analysis based on cp genome sequences showed that *Cynanchum* was closely related with *Asclepias* and *Calotropis.*

*Cynanchum forrestii* Schltr., a medicinal plant belonging to the family Asclepiadaceae, is mainly located in Xizang, Gansu, Sichuan, Guizhou, and Yunnan provinces in China. Root of this species has been widely used as antifebrile, diuretic, and painkiller in traditional Chinese medicine (Chen et al. 1989). However, most of these studies on this species almost focussed on its pharmacological activity, chemical compositions, and quantitative analysis using high-performance liquid chromatography (HPLC) methods, with little involvement in its molecular biology (Liu et al. 2007, 2008). A well-resolved phylogeny based on sufficient molecular markers is essential to understand the relationships among the species of *Cynanchum.* Complete chloroplast (cp) genome sequence could provide valuable data for resolving phylogeny of angiosperms (Ruhlman et al. 2006; Ravi et al. 2008; Lin et al. 2012). Here, we reported the cp genome sequence of *C. forrestii* and revealed its phylogenetic relationships with other species in the family Asclepiadaceae.

In this study, healthy and fresh leaves of *C. forrestii* were sampled from Dali, Yunnan, China (N25°51′48.99″, E100°01′24.04″). The voucher herbarium specimen (No. ZDQ17012) was also collected and deposited into the Herbarium of Medicinal Plants and Crude Drugs of the College of Pharmacy and Chemistry, Dali University. Total DNA was isolated from dried leaf material according to modified cetyltrimethylammonium bromide (CTAB) method (Doyle 1987) and sequenced by next-generation sequencing based on Illumina Hiseq 2500 platform (Novogene, Tianjin, China). The raw data were filtered using Trimmomatic version 0.32 with default settings (Bolger et al. 2014). Then paired-end reads of clean data were assembled into circular contigs using GetOrganelle.py (Jin et al. 2018) with reference (*Cynanchum auriculatum*, accession number: KU900231). Finally, the cpDNA genome was annotated by the Dual Organellar Genome Annotator (DOGMA; http://dogma.ccbb.utexas.edu/) (Wyman et al. 2004) and tRNAscan-SE (Lowe and Chan 2016).

The annotated chloroplast (cp) genome was submitted to the GenBank (No. MN383187). The whole cp genome of *C. forrestii* was 159,917 bp in length and has a typical quadripartite structure, consisting of a large single-copy (LSC) region of 91,189 bp, a small single-copy (SSC) region of 19,972 bp, and two inverted repeat regions (IRa and IRb) of 24,378 bp. The overall GC content of cp genome is 37.8%. The cp genome contained 112 genes, including 78 protein-coding genes, 30 tRNA genes, and 4 rRNA genes. A total of 10 genes contained one intron, and two genes (*clpP* and *ycf3*) contained two introns. All genes occurred as a single copy, except that 18 genes were duplicated in IR regions.

To confirm the phylogenetic position of *C. forrestii*, a total of 18 cp genome sequences were downloaded from the NCBI database. After using MAFFT version 7.149 for aligning (Katoh and Standley 2013), neighbour-joining (NJ) tree was constructed using MEGA X (Kumar et al. 2018) and three species from *Swertia* L. were selected as outgroup. The results showed that *C. auriculatum* was closer to *Cynanchum wilfordii* than *C. forrestii* with a strong support (Figure 1). Moreover, the genus *Cynanchum* L. possessed close phylogenetic relationships with *Calotropis* R. Br. and *Asclepias L*. The cp genome sequence of *C. forrestii* reported in this study might provide useful information for the development of its medicinal value, as well as robust taxonomy and phylogenetic study on *Cynanchum* in the future.

**Figure 1.**
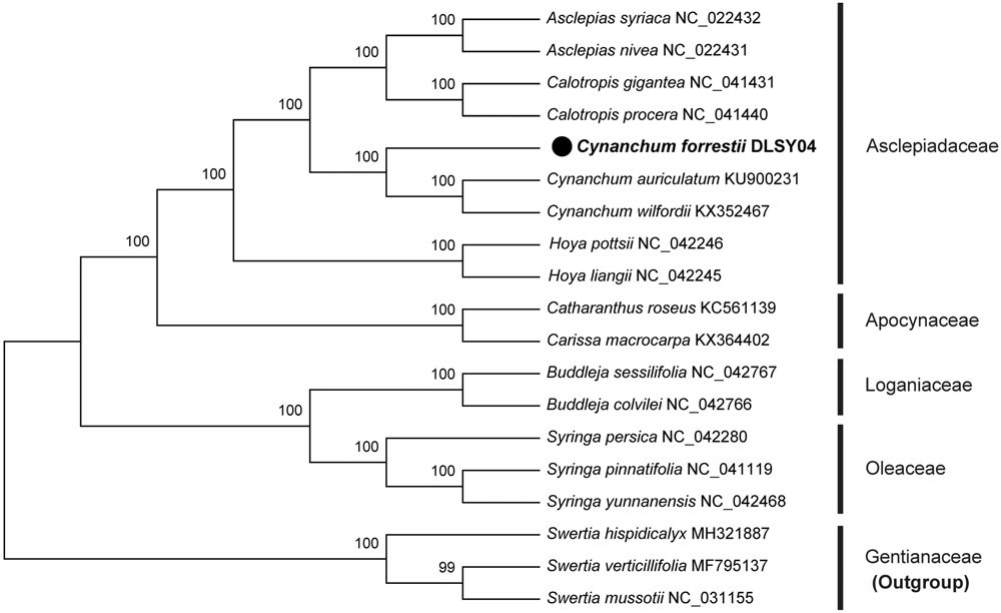
Phylogenetic position of *C. forrestii* inferred by the neighbour-joining (NJ) analysis based on 19 sequences. Numbers in the nodes are the bootstrap values from 1000 replicates.
